# Psychometric Properties of Patient-Facing eHealth Evaluation Measures: Systematic Review and Analysis

**DOI:** 10.2196/jmir.7638

**Published:** 2017-10-11

**Authors:** Bonnie J Wakefield, Carolyn L Turvey, Kim M Nazi, John E Holman, Timothy P Hogan, Stephanie L Shimada, Diana R Kennedy

**Affiliations:** ^1^ The Center for Comprehensive Access and Delivery Research and Evaluation Iowa City Veterans Affairs Healthcare System Iowa City, IA United States; ^2^ Veterans and Consumers Health Informatics Office Veterans Health Administration Washington, DC United States; ^3^ Center for Healthcare Organization and Implementation Research Edith Nourse Rogers Memorial Veterans Affairs Medical Center Boston, MA United States; ^4^ Department of Health Management and Informatics University of Missouri Columbia, MO United States

**Keywords:** telemedicine, computers, evaluation, use-effectiveness, technology, psychometrics

## Abstract

**Background:**

Significant resources are being invested into eHealth technology to improve health care. Few resources have focused on evaluating the impact of use on patient outcomes A standardized set of metrics used across health systems and research will enable aggregation of data to inform improved implementation, clinical practice, and ultimately health outcomes associated with use of patient-facing eHealth technologies.

**Objective:**

The objective of this project was to conduct a systematic review to (1) identify existing instruments for eHealth research and implementation evaluation from the patient’s point of view, (2) characterize measurement components, and (3) assess psychometrics.

**Methods:**

Concepts from existing models and published studies of technology use and adoption were identified and used to inform a search strategy. Search terms were broadly categorized as platforms (eg, email), measurement (eg, survey), function/information use (eg, self-management), health care occupations (eg, nurse), and eHealth/telemedicine (eg, mHealth). A computerized database search was conducted through June 2014. Included articles (1) described development of an instrument, or (2) used an instrument that could be traced back to its original publication, or (3) modified an instrument, and (4) with full text in English language, and (5) focused on the patient perspective on technology, including patient preferences and satisfaction, engagement with technology, usability, competency and fluency with technology, computer literacy, and trust in and acceptance of technology. The review was limited to instruments that reported at least one psychometric property. Excluded were investigator-developed measures, disease-specific assessments delivered via technology or telephone (eg, a cancer-coping measure delivered via computer survey), and measures focused primarily on clinician use (eg, the electronic health record).

**Results:**

The search strategy yielded 47,320 articles. Following elimination of duplicates and non-English language publications (n=14,550) and books (n=27), another 31,647 articles were excluded through review of titles. Following a review of the abstracts of the remaining 1096 articles, 68 were retained for full-text review. Of these, 16 described an instrument and six used an instrument; one instrument was drawn from the GEM database, resulting in 23 articles for inclusion. None included a complete psychometric evaluation. The most frequently assessed property was internal consistency (21/23, 91%). Testing for aspects of validity ranged from 48% (11/23) to 78% (18/23). Approximately half (13/23, 57%) reported how to score the instrument. Only six (26%) assessed the readability of the instrument for end users, although all the measures rely on self-report.

**Conclusions:**

Although most measures identified in this review were published after the year 2000, rapidly changing technology makes instrument development challenging. Platform-agnostic measures need to be developed that focus on concepts important for use of any type of eHealth innovation. At present, there are important gaps in the availability of psychometrically sound measures to evaluate eHealth technologies.

## Introduction

Patient-facing eHealth is a multidisciplinary field focused on the delivery or enhancement of health information and health services through information and communication technologies [1]. eHealth helps consumers engage and collaborate more fully in their health care [2,3], independent of geographic location and also enhances access to health care services by offering novel channels for communication and information flow that complement existing systems [4]. There are many terms related to eHealth, including consumer health informatics, digital health, virtual care, connected care, and telehealth, to list only a few. For purposes of consistency, we use the term “eHealth.”

This paper focuses on patient use of eHealth, which includes personal health records and patient portals accessed via computers or mobile devices, and other telehealth devices designed for use primarily by patients and caregivers, even though some patient-facing technologies (eg, secure patient-provider messaging, mobile apps) are also used by clinicians [5]. Several constructs are important to measure to evaluate patient-facing eHealth technologies. Patient-facing eHealth technologies are used to deliver interventions intended to promote healthy behaviors or effective self-management among consumers. When assessing the efficacy of a behavior-change eHealth intervention, evaluations must address both the intervention and the technology platforms and functions used to deliver the intervention in terms of usability, functionality, and availability of the technology to target users [3]. eHealth may improve the efficiency of and accessibility to clinical and health promotion services for patients. For example, it is anticipated that eHealth may reduce the distance between services and the target user, improving accessibility, or reducing physician or patient workload for a specific task, enhancing efficiency [6-9]. Finally, almost all behavior-change eHealth interventions aim to improve communication in one form or another [10,11].

Although studies using eHealth technologies may include measures that attempt to quantify the characteristics or effect of eHealth interventions, to date, there are no uniform, widely agreed-on measures. More rigorous measurement is needed to determine the full benefit(s) of an eHealth-delivered intervention to both patients and the health care system [12]. Scientific inquiry in other domains has benefited from the development of such standardized measures. At present, various measure compendiums are available that categorize measures of patient-reported outcomes. The Grid-Enabled Measures (GEM) database, for example, was developed starting in 2010 with the purpose of moving social and behavioral science forward by promoting the use of standardized measures tied to theoretically based constructs and facilitating sharing of data from use of standardized measures [13]. Sponsored by the National Cancer Institute, GEM is an open-source measure compendium that solicits scientific community participation in contributing and selecting measures. Users can add information about constructs, find measures related to constructs, upload new measures, provide feedback on existing measures, and search for and share harmonized data for meta-analyses. In addition to providing useful information such as associated references and information on validity and reliability, the GEM allows researchers to see how often other researchers have used a measure and the feedback and ratings they have provided.

Similarly, the Patient-Reported Outcomes Measurement System (PROMIS) was developed by the National Institutes of Health in an effort to develop, validate, and standardize items that may be used to measure patient-reported outcomes common across medical conditions [14]. PROMIS is collecting and testing items focused on patient-reported outcomes of interest, as opposed to validated instruments. For example, the item banks for physical function, fatigue, and sleep disturbance contain 124, 95, and 27 items, respectively [15]. These item banks are being tested in large populations [16-18].

Both PROMIS and GEM promote use of standardized measures and data analysis across multiple studies and conditions. Although these measures can be an important component of studies focused on use of eHealth technologies, the items and instruments contained in these compendiums do not specifically focus on issues surrounding use of eHealth technology with and by patients. For example, although GEM or PROMIS may include instruments or items that measure patient satisfaction with communication with a physician, they do not include items specific to physician-patient communication when using telehealth or secure messaging, nor do they specifically address technology usability issues. Recent efforts to summarize measures related specifically to technology use include a compendium of health information technology-related survey tools developed by the Agency for Healthcare Research and Quality (AHRQ). The AHRQ compendium includes a wide variety of measures, but the website does not provide detailed information on psychometric properties. Thus, although work is in progress to develop and identify measures that may address eHealth evaluation needs, more work is needed.

Implementation research focuses on structural and organizational characteristics of the environment where an innovation is being or will be used. Within this environment are individuals (patients, providers, administrators) with various characteristics that may hinder or facilitate adoption of the innovation within the particular environment. In this review, we focus on the innovation (ie, the eHealth intervention) and how features of this innovation will impact implementation. Consistent and well-validated measures will contribute to determining the true benefit of eHealth interventions across studies and over time. Consistently used measures will enable the health care system to collect uniform data on (1) the likelihood of adoption of an eHealth technology; (2) patient, organizational, or health care system barriers and facilitators to adoption; (3) user attitudes toward and/or satisfaction with a technology; (4) the degree to which meaningful user characteristics (eg, health literacy) mediate the relationship between technology use and improved health outcomes (ie, improved self-management of chronic illness, reduced health care utilization), and (5) the return on investment of eHealth technology to assess value.

The objective of this project was to conduct a systematic review to (1) identify existing instruments for eHealth research and implementation evaluation, (2) characterize measurement components, and (3) assess psychometrics. Additionally, this study seeks to highlight current limitations of this body of research.

## Methods

### Identification of Search Terms

Through a series of investigator meetings, we identified key concepts from existing models, published studies of technology use and adoption, and sociotechnical perspectives on health information technology implementation and evaluation [19-23]. Using these models and studies, our knowledge of the field, and detailed input from an experienced health sciences librarian, we developed a working list of key concepts to focus our search. These were then categorized into five areas: platforms (eg, email), measurement (eg, survey), function/information use (eg, self-management), health care occupations (eg, nurse), and eHealth/telemedicine (eg, mHealth) (Multimedia Appendix 1). Our focus was to identify instruments that could be used for any of these concepts as well as those that may be relevant to only one or two concepts.

### Search Strategy

We conducted a systematic search of the literature using the selected search terms. Based on guidance from our health sciences librarian, databases used included MEDLINE, Scopus, PsychInfo, CINAHL, Health and Psychosocial Instruments (HAPI) for articles published through June 2014. Each database was searched using terms included in Multimedia Appendix 1. The search logic followed this format: (A and D and B and C) OR (E and B and C). All terms listed in sets A, B, D, and C were entered and combined using the Boolean operator “and.” Likewise, terms in sets B, C, and E were entered and combined using “and.” The results from these two searches were then combined using the operator “OR.” This logic was used to ensure all possible terms were included and ensured studies included some sort of measurement or evaluations.

Our search strategy also included review of currently funded research projects within the health services research arm of the Veterans Health Administration (VA) system focused on eHealth (n=56), and existing instrument/measure compendiums (GEM, PROMIS, AHRQ). All search results were transferred to a reference management software database (EndNote); duplicates, articles where the text was not in English, and books were eliminated.

### Inclusion Criteria

Our article inclusion criteria were broad to identify the full extent of instruments designed for eHealth research and implementation evaluation. We focused explicitly on instruments that assessed an eHealth-specific construct from the patient’s point of view. Articles were selected if they (1) described development of an instrument, or (2) used an instrument in an evaluation of an eHealth technology that could be traced back to an original publication describing its development, or (3) modified an instrument, and (4) with full text in English language. The review was limited to instruments that reported at least one psychometric property. Excluded were investigator-developed measures or sets of questions without psychometric evaluation, disease-specific assessments delivered via technology or telephone (eg, a cancer-coping measure delivered via computer survey), and measures focused primarily on clinician use (eg, the electronic health record). We limited our review to articles that reported at least one established psychometric property (see Table 1 for psychometric evaluation components).

### Data Extraction

Two investigators and a research assistant (BW, JH, AM) independently reviewed 100 article titles followed by an in-depth discussion to establish agreement on inclusion of articles. Next, the review was repeated two times using an additional 100 article titles each time, until agreement was reached on articles to include for further review. All article titles were then reviewed to exclude ineligible articles. The abstracts of the remaining articles were reviewed by a pair of investigators (BW, CT) following an independent review of 20 articles to establish interrater consistency. The remaining abstracts were then independently reviewed and discrepancies between reviewers were resolved by discussion and consensus. Articles that did not meet criteria were excluded (no instrument, use of an instrument, or instrument modification), and remaining articles were retained for full-text review. Articles were then classified as describing the development and testing of an instrument or as using an instrument. For articles using an instrument, reference lists were reviewed to identify citations for the original instrument development.

A data extraction form with definitions for each item was developed by the study team (Table 1) [24]. To establish interrater reliability in data extraction, coauthors were divided into pairs, and were assigned to independently review two articles using the data extraction tool. These reviews were discussed in depth by the whole study team to reach consensus on the definitions used in Table 1. Following minor revisions of the data extraction form, articles from the search were then distributed among the six study investigators for final review and data extraction. The first author then reviewed each article and data extraction information to ensure accuracy.

**Table 1:** Data extraction elements.

Element	Definition
Construct	Constructs are not directly observable, but may be applied and defined based on observable behavior; many health measures are designed to capture some aspect of an underlying construct. In the authors’ own words, what the authors of the scale say they are measuring.
Theoretical foundation	Conception of how attributes exist and relate to one another; theoretical framework; can indicate that a conceptual framework (concepts identified in the framework) was used.
Modification of another instrument by others (alternate forms) abbreviated, short forms, different forms targeting the same construct, translations	State if this article is a modification of the format or administration of an instrument already evaluated for psychometric properties.
# items	Number of items included in the measure.
Item types	Structure of the items: such as Likert-type, categorical (multiple options), open ended, yes/no, visual analog scale, other.
Administration time	Estimated amount of time for completion of the measure.
Administration mode	Assessment completed by self-report vs interviewer/researcher administered.
Active vs passive assessment/obtrusiveness	Data collection which does not involve direct solicitation from the research subject or other participant; indirect ways to obtain the necessary data often relying on technology captured information such as response time, number of navigation errors, etc.
Item development	Briefly overview how items were developed for the original form of the measure (ie, expert generation of items, compilation of items from prior measures).
Scoring	Describe how the measure is scored, include a range of possible scores and other descriptive statistics such as significant threshold scores if available.
Readability	Did the developers test the readability of the measure? Were any readability formulas used (eg, Flesch-Kincaid).
Sensitivity to change	Ability to detect change over time, particularly in response to some intervention; known as responsiveness; floor and ceiling effects.
Reliability: test-retest	Consistency in scores between 2 administrations of the measure separated by time (ie, same subject completes the measure twice).
Reliability: interrater	Consistency between 2 independent observers using the measure (for measures that involve observing subjects)% agreement, kappa.
Reliability: internal consistency	Degree to which all items in the scale correlate with each other taking length of measure into account, indicating the items measure the same underlying construct. Based on a single administration of the measure; Cronbach alpha, Kuder-Richardson, split-half reliability.
Validity: content	Typically, from a review of the literature or review by experts.
Validity: criterion, convergent, concurrent, discriminant	Correlation of the scale with other measures to determine independence from other constructs yet some positive correlation to similar constructs and negative correlation to dissimilar constructs.
Validity: construct	Linking the measure to another known attribute. Factor analysis to identify proposed underlying constructs consistent with proposed theoretic content of the measure.
Sample	Patient population used to develop, validate, or test the measure.
Sample studies using the metric/strength of evidence	Studies using the measure including those that did not present psychometric properties of the measure.
Measure website address	If the measure has an associated website, list the website address here and note the date of last update, if available.
Copyright or fees associated with use of the measure	Requires purchase of the measure or the scoring algorithm?

## Results

**Figure 1:**
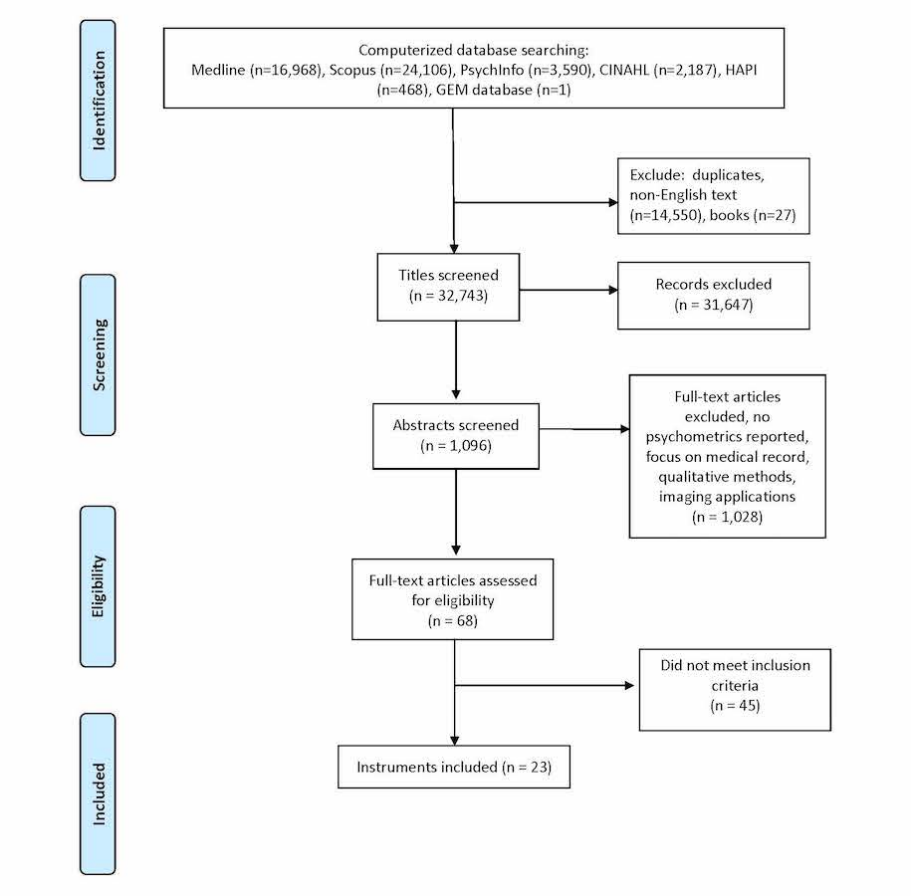
Flow diagram of search.

The search strategy yielded 47,320 articles (PubMed: n=16,968; Scopus: n=24,106; PsychInfo: n=3590; CINAHL: n=2187; HAPI: n=468; GEM: n=1). Following elimination of duplicates and full text not in English language publications (n=14,550) and books (n=27), most articles were excluded through review of titles (n=31,647). Following a review of the abstracts of the remaining 1096 articles, 68 were retained for full-text review. Of these, 16 described an instrument and six used an instrument; one instrument was drawn from the GEM database, resulting in 23 articles for inclusion in the review (Figure 1). Of these 23 articles, seven were modifications of existing instruments. No additional measures were identified through our VA, PROMIS, or AHRQ search. Each article was then reviewed by team members, using the data extraction form (Table 1).

We identified common conceptual threads across the 23 instruments. We reviewed the literature to identify salient concepts and constructs from existing technology use models [19-22,25]. Multiple constructs were identified and terminology varied across models. For example, the Technology Acceptance Model includes 16 constructs in four categories (behavioral intention, perceived usefulness, perceived ease of use, and use behavior). Although terminology varied by author and model, categorizations were inferred and grouped. Twelve concepts emerged from this categorization: clinical content, communication, effectiveness, efficiency, frequency/consistency of use, hardware and software, perceived ease of use, policies and procedures, risk and benefits, user preferences, social influence, and usability. Author definitions guided this categorization. The definition of several of these terms are intuitive (eg, effectiveness), but some are not and are briefly defined here. Efficiency includes the concepts of accuracy, costs, learnability, performance expectations, productivity, quality of use, and workflow. Learnability is an aspect of usability and refers to the ease of learning how to use software. Closely related to learnability is performance expectation, where the end user knows what is expected from them to use the software. Hardware and software aspects include availability, human-computer interface (ie, efficient and desirable interaction between a person and the computer), information display, system maintenance and monitoring, and technical quality. Perceived ease of use incorporates anxiety about and attitude toward using a computer, behavioral intention (the likelihood that an individual will use the computer), computer self-efficacy, engagement, enjoyment, and usefulness.

**Table 2:** Concepts 1 to 6 identified in reviewed instruments (N=23).

Article	Concept and model authors					
	Clinical content [20]	Communication [20,21]	Effectiveness [22]	Efficiency [20-22]	Frequency/consistency of use [21,23]	Hardware and software [19-23]
Atkison, 2007 [29]			**X**	**X**		
Bakken, 2006 [30]			**X**	**X**		**X**
Brockmeyer, 2013 [31]						**X**
Brooke, 1996 [32]			**X**	**X**		
Bunz, 2004 [33]			**X**	**X**	**X**	**X**
Demiris, 2000 [34]			**X**	**X**		**X**
Finkelstein, 2012 [35]	**X**		**X**	**X**	**X**	
Henkemans, 2013 [36]	**X**					**X**
Hudiberg, 1991-1996 [37-40]			**X**	**X**		**X**
Jay & Willis, 1992 [41]			**X**			
Lewis, 1993 [42]			**X**	**X**		**X**
Lin, 2011 [43]				**X**		**X**
Martinez-Caro, 2013 [44]	**X**				**X**	**X**
Montague, 2012 [45]	**X**		**X**	**X**		**X**
Norman, 2006 [46]	**X**		**X**	**X**	**X**	**X**
Pluye, 2014 [47]	**X**	**X**		**X**	**X**	**X**
Schnall, 2011 [48]	**X**	**X**	**X**	**X**		
Tariman, 2011 [49]	**X**		**X**	**X**		**X**
Wang, 2008 [50]	**X**		**X**	**X**		**X**
Wehmeyer, 2008 [27]						**X**
Wolfradt, 2001 [51]		**X**				**X**
Xie, 2013 [28]	**X**		**X**	**X**		**X**
Yip, 2003 [52]	**X**					**X**

The 23 articles included in this review were mapped to the 12 identified concepts based on whether the instrument encompassed the concept. The most common constructs addressed by this set of measures were effectiveness, efficiency, hardware and software, perceived ease of use, satisfaction, and usability [19-23] (Tables 2 and 3). Interestingly, although eHealth is a communication technology, only three studies specifically address this aspect. Additionally, to identify potential gaps for future consideration, concepts included in the measures, but not identified in the 12 model concepts, were documented in the crosswalk (last column in Table 3). For example, stress, eHealth literacy, perceived necessity, and others emerged as concepts not identified in the review of existing technology use models. eHealth literacy is defined by Norman and Skinner [26] as “the ability to seek, find, understand, and appraise health information from electronic sources and apply the knowledge gained to addressing or solving a health problem.” Wehmeyer [27] introduced three concepts: symbolism, esthetics, and perceived necessity. Symbolism reflects the meaning or status associated with the device (eg, having a mobile device may signify group membership or a certain social status). Esthetics refers to the appearance of the device (eg, the perceived beauty of the device may affect the attachment to the device). Finally, the perceived necessity of the device may affect attachment to the device, creating anxiety when the device is not accessible. Xie et al [28] addressed decision-making autonomy, defined as the level of decision making desired when information about health conditions is electronically available.

No instrument included a complete psychometric evaluation (Multimedia Appendix 2). The most frequently assessed property was internal consistency (21/23, 91%). None of the measures were assessed for sensitivity to change, but several authors indicated the instrument was not designed to assess change. Few measures were assessed for test-retest reliability (4/23, 17%) and only one instrument had been tested for interrater reliability. Testing for aspects of validity ranged from 48% (11/23) of measures tested for criterion, convergent, concurrent, or discriminant validity to 78% (18/23) reporting establishing content validity. Approximately half (13/23, 57%) reported how to score the instrument. Only six (26%) assessed the readability of the instrument for end users, although all measures rely on patient self-report.

**Table 3:** Concepts 7 to 12 identified in reviewed instruments (N=23).

Article	Concepts and model authors						Concepts not included in models
	Perceived ease of use [19,21-23]	Policies & procedures [20]	Risk & benefits [23]	Satisfaction/ acceptability/ preferences [23]	Social influence [21]	Usability [23]	
Atkison, 2007 [29]	**X**					**X**	
Bakken, 2006 [30]	**X**			**X**		**X**	
Brockmeyer, 2013 [31]	**X**		**X**	**X**			
Brooke, 1996 [32]				**X**		**X**	
Bunz, 2004 [33]	**X**			**X**		**X**	
Demiris, 2000 [34]	**X**		**X**	**X**		**X**	
Finkelstein, 2012 [35]	**X**			**X**			
Henkemans, 2013 [36]	**X**		**X**	**X**		**X**	
Hudiberg, 1991-1996 [37-40]	**X**		**X**	**X**	**X**		Stress
Jay & Willis, 1992 [41]	**X**				**X**	**X**	
Lewis, 1993 [42]	**X**		**X**	**X**		**X**	
Lin, 2011 [43]	**X**			**X**		**X**	
Martinez-Caro, 2013 [44]	**X**		**X**	**X**			
Montague, 2012 [45]	**X**		**X**	**X**		**X**	
Norman, 2006 [46]	**X**			**X**			eHealth literacy
Pluye, 2014 [47]	**X**			**X**			
Schnall, 2011 [48]	**X**		**X**				
Tariman, 2011 [49]	**X**			**X**			
Wang, 2008 [50]	**X**			**X**		**X**	
Wehmeyer, 2008 [27]	**X**			**X**			Symbolism; esthetics; perceived necessity
Wolfradt, 2001 [51]				**X**			
Xie, 2013 [28]	**X**			**X**		**X**	Decision-making autonomy
Yip, 2003 [52]				**X**			

Early instruments (prior to the year 2000) [32,37-42] focused on using a computer, reflecting early consumer adoption of personal computers. These measures are not specifically focused on “health” use. During the decade from 2000 to 2009, measures that focused on use of information technology related to health began to emerge, focusing primarily on telehealth [30,34,52]; other measures focused on eHealth literacy [46] and use of eHealth education [29]. Other concepts for which measures were developed included using the Internet [51], use of computers [33], use of mobile devices [27,50], and the effect of video games on engagement [31], although these measures did not specifically focus on “health.” Since 2010, the frequency of “health” themes increased including communication between patients and providers [47,49], patient trust [45], preferences [28], satisfaction [35], and use of technology for care provision [48] or patient self-management [36,48]. One instrument also focused more generally on use of computers [43], and one focused on patient loyalty to online services [44].

## Discussion

### Principal Findings

Of the 23 articles reviewed, no instrument included a complete psychometric evaluation. The most frequently assessed property was internal consistency. Testing for aspects of validity ranged from 48% (11/23) to 78% (18/23). Approximately half (13/23, 57%) reported how to score the instrument. Only six (26%) assessed the readability of the instrument for end users, although all the measures rely on self-report.

Common theoretical concepts addressed in the instruments were effectiveness, efficiency, hardware/software, perceived ease of use, and satisfaction. A notable exception is that only three instruments focused on communication. Conversely, we identified some concepts addressed in the instruments that have not been included in current theoretical models, including stress, esthetics, eHealth literacy, comfort, and decision-making autonomy. Current instruments require fuller evaluation of psychometric properties.

Measures that can be applied consistently across technologies and platforms are needed so that distinct platforms that serve the same purpose can be compared. For example, evaluation of an intervention to treat depression could utilize a standard measure of usability, regardless of whether it was a mobile app or Web-based (eg, “It took many tries before I knew how to use the key features of this technology” and “I found the layout of the features very intuitive”), regardless of the platform used to deliver the intervention (eg, mobile app or online program). Using these types of measures, investigators and others implementing eHealth technologies can compare technologies and use this information when selecting a technology.

Our review expands on the AHRQ compendium, which lists available measures but provides less detail about their other attributes. We also investigated whether the psychometric properties of the measures had been established, which is a critical information need when selecting a measure for research or evaluation. However, although most would agree that instruments with psychometric properties are very helpful, there may also be a role for using self-developed questions that may more clearly and directly get at the target construct or a specific patient behavior. The AHRQ compendium is populated with many such instruments and future researchers should carefully consider the trade-offs of using investigator-developed question sets that may specifically address their question of interest versus a more validated instrument that may also need to be modified to fit an eHealth evaluation. Furthermore, investigators may want to consider instruments listed in the AHRQ compendium for further development and psychometric evaluation.

Implementation of eHealth technologies can involve substantial investment in terms of costs and effort. Research on eHealth has also increased dramatically over the past several years, yet studies rarely utilize common methods and/or instruments. The results of this project provide critical insights regarding existing eHealth instruments and identify gaps for which new instruments are needed. Use of common and psychometrically sound instruments can inform future studies so that the results from multiple studies can be compared and synthesized.

Although most the instruments identified in this review were published after the year 2000, rapidly changing technology makes instrument development challenging. Platform-agnostic measures need to be developed that focus on concepts important for use of any type of eHealth innovation. Instrument development as a research enterprise is typically undervalued, relative to more direct practice-relevant research. Instrument development can also be a complex and lengthy process. Thus, funding agencies should consider addressing this gap, given the persistent and expected growth in the deployment of technology to improve care processes and patient outcomes.

### Limitations

We did not conduct a comprehensive search for all published uses of the identified instruments as it was beyond the scope of this study. The grey literature (eg, conference abstracts, dissertations, and unpublished studies) were not included in our review. Furthermore, the review potentially missed some published as well as unpublished measures based on keyword choice and/or elimination of articles through review of title or abstract. Finally, our choice of theoretical models used to analyze the selected articles may impose limitations on our findings.

### Conclusions

Based on our review, we highlight some of the more useful measures that we believe could be useful in most technology studies. These include the eHealth literacy scale (eHEALS) [46], the Computer-Email-Web Fluency Scale [33], and the System Usability Scale [32]. Additional research is needed to build and further refine measures of literacy such as the eHEALS or Computer-Email-Web Fluency Scale so that researchers have access to a validated measure of user’s comfort with a target technology.

Development of a standard measure of the intuitiveness of the user interface would allow platform-agnostic comparisons between user interfaces (eg, two mobile apps for depression, or comparison of differences between a Web-based and mobile app). Finally, given the explosion of new technologies in the market focused on health behaviors, a standard measure of the relative advantage of a new technology feature when compared to prior methods and/or a standard measure of the degree to which new technology facilitates a target behavior (eg, weight loss, exercise, self-management techniques, or receipt of care) could provide important insights to inform technology adoption strategies.

Advances in eHealth offer tremendous potential to improve access to care, efficiency of care delivery processes, and overall quality. Significant resources are being invested in eHealth technologies, driven in part by meaningful use requirements. Consumer behavioral health interventions are increasingly being made available via multiple platforms (eg, computer vs mobile versions of interventions proven effective for in-person delivery). Identification of useful and valid measures to evaluate these interventions has important potential to contribute to improved implementation, clinical practice, and ultimately population health since insights gleaned from standardized measurement can directly inform system improvements and optimal implementation strategies. In addition, having better measures to evaluate implementation of eHealth technologies will help improve consumers’ experiences with technologies and assess whether use of these technologies is making a measurable difference in quality of care or the patient experience. More longitudinal research will be needed to develop measures that more comprehensively address the wider frame of concepts important for the meaningful implementation of eHealth technologies.

## Appendix

Multimedia Appendix 1 Search Terms.

Multimedia Appendix 2 Detailed Psychometric Properties of Reviewed Instruments.

## Abbreviations

AHRQ: Agency for Healthcare Research and Quality
CINAHL: Cumulative Index to Nursing and Allied Health Literature
GEM: Grid-Enabled Measures
HAPI: Health and Psychosocial Instruments
PROMIS: Patient-Reported Outcomes Measurement System
VA: Veterans Health Administration
