# Supplementary material for: Psychometric Properties of Patient-Facing eHealth Evaluation Measures: Systematic Review and Analysis
Source: J Med Internet Res. 2017 Oct 11;19(10):e346. doi: 10.2196/jmir.7638 (PMC5656774; doi:10.2196/jmir.7638)
Supplement: Multimedia Appendix 1 [file jmir_v19i10e346_app1.pdf]

**Set A: Platforms**	**Location**
Cell phone	Title/Abstract
Computer	Title/Abstract
Mobile phone	Title/Abstract
Digital health	Title/Abstract
Electronic communication*	Title/Abstract
Email	Title/Abstract
“Electronic mail”	MESH
Gamification	Title/Abstract
Gaming	Title/Abstract
“Video games”	MESH
Interactive voice response	Title/Abstract
Internet	Title/Abstract
Kiosk	Title/Abstract
Mobile application*	Title/Abstract
Online communit*	Title/Abstract
Online forum*	Title/Abstract
Online support group*	Title/Abstract
Patient portal*	Title/Abstract
Personal digital assistant*	Title/Abstract
Remote monitoring	Title/Abstract
Secure Messaging	Title/Abstract
Smart phone*	Title/Abstract
Social networking	MESH
Social network*	Title/Abstract
Telecommunication	Title/Abstract
“Telecommunications”	MESH
Telemonitoring	Title/Abstract
Telephone	Title/Abstract
Text messaging	Title/Abstract
Texting	Title/Abstract
SMS	Title/Abstract
User interface	Title/Abstract
Video conferencing	Title/Abstract
Web	Title/Abstract
Wireless	Title/Abstract
“Wireless technology”	MESH
World wide web	Title/Abstract
“Computers, handheld”	MESH
“Cellular Phone”	MESH
“computers”	MESH
“Internet”	MESH
“telephone”	MESH
“text messaging”	MESH
“online systems”	MESH

**SET B: MEASUREMENT**	

Instrument	Title/Abstract
Interviews	Title/Abstract
interview	Title/Abstract
Measure	Title/Abstract
“Outcome assessment (health care)”	MESH
“Process assessment (health care)”	MESH
Psychometrics	Title/Abstract
Psychometric	Title/Abstract
Questionnaire	Title/Abstract
Reliability	Title/Abstract
“Reproducibility of results”	MESH
Scale	Title/Abstract
Survey	Title/Abstract
Tool	Title/Abstract
“Validation studies”	Publication Type
Validation	Title/Abstract
Validity	Title/Abstract
“Questionnaires”	MESH
“Evaluation studies as topic”	MESH
“Evaluation studies”	Publication Type

**SET C: FUNCTIONS/ INFORMATION USE**	
“computer literacy”	Title/Abstract
consumer health informatics	Title/Abstract
“care coordination”	Title/Abstract
eHealth literacy	Title/Abstract
e-health literacy	Title/Abstract
information seeking	Title/Abstract
“health literacy”	MESH
heuristics	Title/Abstract
human computer interaction	Title/Abstract
“information literacy”	MESH
“meaningful use”	MESH
participatory design	Title/Abstract
patient access	Title/Abstract
patient activation	Title/Abstract
patient engagement	Title/Abstract
patient-provider communication	Title/Abstract
Doctor-patient communication	Title/Abstract
personal health information management	Title/Abstract
self-management;	Title/Abstract
self management	Title/Abstract
“social support”	MESH
usability	Title/Abstract
user centered design	Title/Abstract
user-centered design	Title/Abstract
“case management”	MESH
“user-computer interface”	MESH

“consumer participation”	MESH
“patient access to records”	MESH
“health communication”	MESH
“health information management”	MESH
“self care”	MESH
case management	MESH
“information seeking behavior”	MESH
“attitude to computers”	MESH
“patient satisfaction”	MESH
“patient preference”	MESH
“patient acceptance of health care”	MESH
“health promotion”	MESH

**SET D: Healthcare Occupations**	
Health*	Title/Abstract
Medicine	Title/Abstract
Nurse*	Title/Abstract
Dentis*	Title/Abstract
Psychiatr*	Title/Abstract
Pharmacist*	Title/Abstract
Pharmacy	Title/Abstract
Social work*	Title/Abstract
“Health occupations”	MESH
“Delivery of healthcare”	MESH
Psychology*	Title/Abstract
Medical	Title/Abstract
Nursing	Title/Abstract

**SET E: eHealth/Telemedicine**	
eHealth	Title/Abstract
e-health	Title/Abstract
mHealth	Title/Abstract
m-health	Title/Abstract
“Health records, personal”	MESH
Telehealth	Title/Abstract
Telemedicine	Title/Abstract
Telepatholog*	Title/Abstract
Teleradiolog*	Title/Abstract
Telenurs*	Title/Abstract
Teledentist*	Title/Abstract
“Telemedicine”	MESH
“Therapy, computer-assisted”	MESH
