# Supplementary material for: Psychometric Properties of Patient-Facing eHealth Evaluation Measures: Systematic Review and Analysis
Source: J Med Internet Res. 2017 Oct 11;19(10):e346. doi: 10.2196/jmir.7638 (PMC5656774; doi:10.2196/jmir.7638)
Supplement: Multimedia Appendix 2 [file jmir_v19i10e346_app2.pdf]

| **Citation** | **Atkinson, 2007** | **Bakken et al., 2006** | **Brockmyer, et al., 2009** |
|---|---|---|---|
| Instrument name | No name | No name | Game Engagement Questionnaire |
| Construct | Perceived characteristics of an eHealth education innovation | Satisfaction with telemedicine | Engagement of players during violent video games; constructs examined include flow and psychological absorption |
| Theoretical foundation | Diffusion of innovations | NR | Rasch approach to measure development |
| Modification of another instrument | Yes | NR | No |
| # items | 30 | 21 | NR |
| Item types | Likert-type | Likert-type | yes/no questions |
| Administration Time | NR | NR | NR |
| Item development | Re-worded items from a previous study by Bolton (1983) | Selected 51 items from other surveys; telemedicine experts prioritized items & suggested new ones; developed English and Spanish versions | 1) Existing measures were reviewed, 2) Focus groups were conducted with children and adults, 3) the measure was administered to different groups of players<br><br>Item development focused on the participant's tendency to become engaged in violent video games |
| Scoring | NR | | NR |
| Readability | NR | 8th grade (range across items was 2.2 to 12th grade) | NR |
| Sensitivity to change | NR | NR | NR |
| Reliability: test-retest | NR | NR | NR |
| Reliability: inter-rater | NR | NR | NR |
| Reliability: internal consistency | Items with Cronbach’s alpha ≥0.70 retained | Video Visits Cronbach’s alpha 0.96; 0.92 for Use & Impact | NR |

| | | | |
|---|---|---|---|
| Validity: content | NR | Review by four telemedicine experts | Reviewed existing measures of related constructs and conducted focus groups for item construction. |
| Validity: criterion, convergent, concurrent, discriminant | NR | Significant discriminant validity between Hispanics and non-Hispanics | NR |
| Validity: construct | Confirmatory and exploratory factor analyses to evaluate a priori scales consistent with diffusion of innovation framework | Factor analysis resulted in two components: Video Visits and Use & Impact | NR |
| Sample | College students | Home based telemedicine intervention participants with diabetes | Junior high school students age<br>12 to 15 years' male undergraduates attending a midsized Midwestern university |
| Limitations | Use of a convenience sample, small sample size, one wave of data collection | Used different methods of administration in subgroups, i.e., mailed surveys vs. interviews Items with higher readability levels included words such as telemedicine, blood pressure testing, blood sugar testing, ADA educational web pages, and privacy. | If expanded to include non-violent video games, additional items would need to be included |

| **Citation** | **Brooke, 1996** | **Bunz, 2004** | **Demiris et el., 2000** |
|---|---|---|---|
| Instrument name | System Usability Scale | Computer-email-web Fluency Scale | Telemedicine Patient Satisfaction Questionnaire |
| Construct | Usability | Computer and email fluency, Web navigation and editing | Consumer perception of the risks and benefits of home telecare |
| Theoretical foundation | NR | NR | NR |
| Modification of another instrument | NA | Yes | NR |
| # items | 10 | 21 | 17 |

| | | | |
|---|---|---|---|
| Item types | Likert-type | Likert-type | Likert-type |
| Administration Time | NR | NR | NR |
| Item development | 20 participants rated two software systems (one rated easy to use, the other rated difficult to use) using an initial pool of 50 items. 10 items that led to the most extreme responses were selected for the final scale | Original 46 items- 32 participants identified confusing questions and possible content areas that were missing. This yielded 52 revised items then used in scale development. Final scale after reliability and validity studies yielded 21-item measure. | Items were selected from a review of literature and focus group discussions |
| Scoring | The score contribution for items 1,3,5,7 and 9 is the scale position minus 1; the score contribution for items 2, 4, 6, 8 and 10 is 5 minus the scale score. Scale contributions are summed, then multiplied by 2.5; scores have a range of 0 to 100. | Likert scale item summed | Responses based on 5-point Likert scale with overall scores ranging from 17 to 85; higher score indicates a more positive patient perception of telehomecare. |
| Readability | NR | NR | NR |
| Sensitivity to change | NR | NR | NR |
| Reliability: test-retest | NR | NR | The control group did not have a statistically significant change in overall scores after a 30-day period (mean difference=0.18, t=0.69, p=0.51). Also, there was no significant change in score for each item for the pre- and post-test in the control group. |
| Reliability: inter-rater | NR | NR | NR |
| Reliability: internal consistency | Intercorrelations of items ranging from ± 0.7 to ± 0.9 | Total scale coefficient alpha=0.89; computer subscale= 0.72. Email Subscale=0.75, Web Navigation =0.64., web editing 0.79. | Cronbach's alpha 0.8 |

| | | | |
|---|---|---|---|
| Validity: content | Software systems rated by users in the scale development process were generally agreed to be “really easy to use” or “almost impossible to use.” | Reviewed by students for question design and presence or absence of key concepts. | Results from 9 focus groups and feedback from telemedicine researchers |
| Validity: criterion, convergent, concurrent, discriminant | NR | NR | NR |
| Validity: construct | In scale development, 10 items (from a pool of 50) that elicited the most extreme responses when rating an easy-to-use and a hard-to-use software system of 50 were selected | Conducted a principal component, varimax rotation, factor analysis which yielded four factors: Basic Computer Skills, Basic e-Mail scales, Web navigation, and Web editing. Total percent of variance accounted for was 67.3%.<br>The CEW showed adequate independence from other measures of computer use and satisfaction such as the Computer Use Scale, (Panero, Lane, & Napier, 1997) frequency of use, comfort with computer use, number of computer courses taken. Duration of Internet Usage and Level of Expertise predicted CEW fluency total score (R-square=0.368). | High correlation between similarly worded items |
| Sample | Office systems engineering group | College students | Hospital patients (and some of their carers) who were about to be discharged to home care, home care patients in assisted-living facilities and members of a cardiac rehabilitation support group |

| | | | |
|---|---|---|---|
| Limitations | Does not identify what makes a system usable (or not) | Convenience sample of college students<br><br>Author acknowledges that scale might be improved by including items with more advanced skills. | Sample size; severity of disease was not included in analysis |

| **Citation** | **Finkelstein, et al. , 2012** | **Henkemans et al. , 2013** | **Hudiburg, 1989, 1989, 1993, 1995** |
|---|---|---|---|
| Instrument name | No name | eHealth Analysis and Steering Instrument | Computer Technology Hassles Scale |
| Construct | Patient satisfaction | 3 dimensions that contribute to the effectiveness of eHealth supporting self-management: 1. Utility 2.Usability<br>3. Content | Computer-related stress independent of attitudes toward computer technology. |
| Theoretical foundation | NR | NR | NR |
| Modification of another instrument | NR | NA | NA |
| # items | 15 | 32 | 37 |
| Item types | Likert-type and 3 open ended items | Dichotomous items (applicable, not applicable) | Severity rating scale |
| Administration Time | NR | NR | NR |
| Item development | Compiled from a literature review and other surveys and item banks | Generated 43-items to reflect the three concepts described above. | Patterned after the Daily Hassles Scale |
| Scoring | Five point Likert scale; scoring range 15 to 75 | 0 or 1 for each item which is summed for the total score | The number of hassles endorsed is counted with a total score ranging from 0 to 69. A second way to score is deriving a total severity score by adding the severity for all the items checked, with a possible range from 0 to 207. The two types of scoring are highly correlated. |

| | | | |
|---|---|---|---|
| Readability | Sixth grade based on Flesch-Kincaid Readability Index | NR | NR |
| Sensitivity to change | NR | Construct not expected to change. | Not necessarily expected to change. |
| Reliability: test-retest | Testing approximately 2.5 months apart; intra-class correlation coefficient was 0.77 | NR | Test-Retest coefficient = 0.64 over a two-month time period. |
| Reliability: inter-rater | NR | Inter-rater reliability for items ranged from Kappa=0.41 to Kappa= 0.81. Three items showed perfect agreement. | NR |
| Reliability: internal consistency | Cronbach's alpha was 0.93 overall | Cronbachs alpha for three dimensions ranged between 0.56-0.62. | Coefficient alpha=0.95 for the full scale and 0.94 for the Computer Runtime Errors subscale and 0.89 for the Computer Information Problems subscale. |
| Validity: content | Based on sources of items, judgment of research team, and informal discussions with study participants | Face validity was tested through a Delphi process using Dutch experts. This process eliminated 8 items and retained 35. Both exploratory and confirmatory factor analysis yielded a one-factor solution. | NR |

| | | | |
|---|---|---|---|
| Validity: criterion, convergent, concurrent, discriminant | Criterion assessed by correlation between actual home monitoring adherence and self-reported adherence (0.87) | Determined the degree to which ratings actually predicted outcomes of RCTs that already occurred. RCT outcomes were put in categories: determinants of behavior, self-management behavior, health outcomes.<br><br>Total eASI predicted 31% of the variance in the effect sizes of selected RCTs on self-management behavior. Usability and Utility subscales also predicted effectiveness on self-management behavior. Total eASI score did not predict effectiveness on health outcome measures. Usability predicted 13% of the variance on health outcomes, but the other two subscales were not predictive. | Computer Technology Hassles Scale correlated positively with the Perceived Stress Scale (r=0.26) but was not related to the Computer Attitude Scale. The Computer Hassles Scale correlated with the Symptom Checklist – 90 (r=0.34, p<0.01). |
| Validity: construct | NR | NR | Factor analysis confirmed a four factor model, in contrast to the two-factor model assumed by the two subscales. |
| Sample | Patients participating in home spirometry monitoring | 7 men and 9 women age 20 to 25 years | College age students. |

| | | | |
|---|---|---|---|
| Limitations | Limited sample size | Authors acknowledge the reliability and validity could be improved upon. They recommend using a Likert type of response rather than dichotomous ratings. They also recommend some content changes for the items. Authors state they have a small sample of raters and did not use a large sample of RCTs. Moreover, the RCTs where predominantly European. | Needs to be validated in other populations; older instrument |

| **Citation** | **Jay & Willis 1992** | **Lewis, 1995** | **Lin, 2011** |
|---|---|---|---|
| Instrument name | No name | Computer Usability Satisfaction Questionnaire | No Name |
| Construct | Seven dimensions of attitudes toward computers: comfort, efficacy, gender equality, control, dehumanization, interest, and utility | User satisfaction with computer system usability. | Computer literacy defined as "basic computer skills, whereas computer competency is defined as the computer skills necessary to accomplish a job task." |
| Theoretical foundation | NR | NR | NR |
| Modification of another instrument | NA | NA | NA |
| # items | 32 | 19 | 22 |
| Item types | Likert items across 7 factors | Likert-type scale | Likert-type |
| Administration Time | NR | NR | NR |

| | | | |
|---|---|---|---|
| Item development | Compiled from a literature review | The PSSUQ, the measure that preceded the CSUQ, was created by IBM usability experts using a list of usability attributes "known to influence user perception of usability….a series of investigations using decision support systems revealed a common set of five system characteristics associated with usability by several different user groups." | The scale developed in this study is based on the Ministry of Education course guidelines and relevant literatures on computer literacy and computer competency." The researchers narrowed the questionnaire to six constructs (domains) and their corresponding measurement items based on the literature. |
| Scoring | Factor scores calculated for 7 factors, standardized to a mean of 0 and SD of 1 | Average the scores from items to obtain the scale and subscale scores. Low scores are better | NR |
| Readability | NR | NR | NR |
| Sensitivity to change | NR | NR | NR |
| Reliability: test-retest | NR | NR | NR |
| Reliability: inter-rater | NR | NR | NR |
| Reliability: internal consistency | Cronbach's alpha across 7 factors ranged from .54 to .82 | Coefficient alpha exceeded 0.89, indicating acceptable scale reliability. | Cronbach's alpha values for the six constructs were .828, .867, .932, .838, .894, and .848, respectively; overall scale reliability was .923 |

| | | | |
|---|---|---|---|
| Validity: content | 7-factor structure developed with college students and confirmed in an elderly sample | Items were developed by IBM usability experts using a list of usability attributes known to influence user perception of usability. | Eleven experts reviewed content relevance; A content validity index between .6 and .8 was considered acceptable but requiring modification based on experts' suggestions. Of the 24 items, all scored higher than .82, except for Multimedia question 3, which scored .73. After adjustment of MM3, this study had a total of 24 measurement items. After an exploratory factor analysis, the measure was narrowed down to 22 items. |
| Validity: criterion, convergent, concurrent, discriminant | NR | NR | Studied factor loadings (criterion > 0.7), Composite reliability values and AVE. Factor loadings for all items and CR values for all constructs were above .7. Average variance expected values for all constructs were above .5 (all values were above recommended levels). |
| Validity: construct | NR | NR | NR |
| Sample | Developed with college students and validated in an elderly sample | Employees of temporary health agencies with at least 3 months experience with a computer | Nursing students, and Exercise & Health Science department students; 79% were women. |

| | | | |
|---|---|---|---|
| Limitations | 73% of the elderly validation sample were educated women; only short term training effects were measured (2 weeks) | The article described multiple questionnaires (i.e., PSSUQ, ASQ, CSUQ) and concluded that a limitation with all the tests were that the validity measures were all concurrent and that future research should measure the predictive validity. The authors indicate that further research should utilize available scales to construct a “multitrait-multimethod matrix to investigate convergent and discriminant validity for the construct of usability.” | Needs to be assessed with other populations. |

| **Citation** | **Martinez-Caro et al., 2013** | **Montague & Osan, 2010** | **Norman & Skinner, 2006** |
|---|---|---|---|
| Instrument name | e-Loyalty Scale | Trust in Medical Technology | eHEALS |
| Construct | e-Loyalty toward online health care services | Patients’ trust in technologies used in their care across three dimensions (technology, care provider and how the provider uses technology) | Ehealth literacy - the ability to seek out, find, evaluate and appraise, integrate, and apply what is gained in electronic environments toward solving a health problem |
| Theoretical foundation | Marketing models, Technology Acceptance Model combined with satisfaction | NR | Based on Norman and Skinner’s eHealth Literacy Model which includes six core skills, or literacies: (1) traditional literacy, (2) health literacy, (3) information literacy, (4) scientific literacy, (5) media literacy, and (6) computer literacy |
| Modification of another instrument | No | NA | NA |

| | | | |
|---|---|---|---|
| # items | 15 | 80 | 8 |
| Item types | Likert-type scale | NR | Likert scale |
| Administration Time | 10 minutes | NR | NR |
| Item development | Items developed to reflect Perceived Ease of Use, Perceived Usefulness, Attitude, Satisfaction, and e-Loyalty intent. Some adapted from prior work | Based on a thesis completed in 2008 http://scholar.lib.vt.edu/theses/available/etd-04072008-233305/unrestricted/01Abstractrevised.pdf | Items created following a review of the literature and existing measures pertaining to the six core literacies noted above. Pilot testing of items completed with a sample of 89 youth ages 18-24. Based on feedback on readability, item wording the eHEALS was reviewed and modified to create the current 8-item scale.<br>664 participants age 13-21 completed 8-item eHEALS at for time points over 6 months to psychometrically test eHEALS. |
| Scoring | 1 to 7 for each item relating to anchors of the 7-point Likert | NR | NR |
| Readability | NR | NR | NR |
| Sensitivity to change | Not meant to assess change. | NR | NR |
| Reliability: test-retest | NR | NR | Baseline to 6-month follow-up (*r* = .49 to .68); intra-class correlation between different scores .49 |
| Reliability: inter-rater | NR | NR | NR |

| | | | |
|---|---|---|---|
| Reliability: internal consistency | Scale Composite Reliability ranged from 0.83 – 0.85 for 5 subscales. Cronbach's alpha ranged from 0.80-0.85 for subscales. | NR | Item-scale correlations between items ranged from *r* = .51 to .76. Principal components analysis was performed and produced a single factor solution as expected (eigenvalue = 4.479, 56% of the variance explained). Factor loadings ranged from .60 to .84 among the 8 items; coefficient alpha α=.88 |
| Validity: content | Confirmatory factor analysis validated subscales | NR | NR |
| Validity: criterion, convergent, concurrent, discriminant | Conducted structural model equations between subscales. | NR | NR |
| Validity: construct | Discriminant validity found between constructs demonstrating their independence | NR | No relationship to gender, age, self evaluation of health, use of information technology overall, or type of technology used |
| Sample | Users of online health services ranging in age from18 to 81 years (mean 33.6); 71% had college degrees | Obstetric patients | Ethnically diverse adolescents ranging in age from 13 to 21 years enrolled in secondary school in Canada |
| Limitations | Scale validated in a restricted geographic area- Murcia Spain. Reliance of self-report for both measure and its validators may be biased by response distortions. | Sample limited to obstetric patients; unable to calculate a response rate due to use of email survey; needs to ba validated for a variety of technologies | Lower than expected test-retest reliability correlations; measures consumers perceived skills, not actual skills |

| **Citation** | **Pluye et al. 2014** | **Schnall & Bakken, 2011** | **Tariman et al., 2011** |
|---|---|---|---|

| | | | |
|---|---|---|---|
| Instrument name | Information Assessment Method | No name | Acceptability e-Scale |
| Construct | Knowledge translation between information providers and consumers | Technology acceptance | Acceptability of online assessment of symptom or quality of life screening for cancer patents |
| Theoretical foundation | Acquisition cognition – Level of Outcomes Model | Technology Acceptance Model | NR |
| Modification of another instrument | No | NA | Yes |
| # items | 19 | 9 | 6 |
| Item types | Yes/no | Likert-type scale | Likert-type |
| Administration Time | NR | NR | NR |
| Item development | Review of literature and prior work (Pluye et al. Four levels of outcomes of information-seeking: a mixed methods study in primary health care. J Am Soc Inf Sci Tec. 2012;64(1):108–125. doi: 10.1002/asi.22793) Interviews with laypersons<br>Expert panel review | Items were developed based on constructs in the TAM and adapted from existing questionnaires. | Items selected from the 10-item post-survey patient impression Form developed by Carlson et al. (2001) and by Taenzer et al. (1997). |
| Scoring | NR | Responses to structured survey items are summed. Two items are negatively worded and reverse coded so that a higher score was associated with more positive attitudes towards the system. | 1 to 5 for each Likert item; 80% of total possible score or higher considered “Acceptable”; no validation of this threshold provided. |
| Readability | NR | NR | Fifth-grade Level. No item was more than 14 words or more than 18 syllables. |
| Sensitivity to change | NR | NR | Not meant to assess change. |
| Reliability: test-retest | NR | NR | NR |

| | | | |
|---|---|---|---|
| Reliability: inter-rater | NR | NR | NR |
| Reliability: internal consistency | NR | (a) Perceived Usefulness (3 items, Cronbach's alpha=0.91), (b) Perceived Ease of Use (3 items, Cronbach's alpha=0.89) and (c) Perceived Barriers to Use (2 items, Cronbach's alpha=0.69). | Coefficient alpha = 0.757; Item to total scale correlation: range 0.312-0.715 with only two below 0.40. Coefficient alpha =0.721. Item total scale correlations ranging from 0.211-0.663 with only two falling below 0.40 |
| Validity: content | Review of literature and prior work; Interviews with laypersons; Expert panel review | Extensive review of the literature | NR |
| Validity: criterion, convergent, concurrent, discriminant | NR | NR | NR |
| Validity: construct | NR | Principal component factor analysis with Varimax rotation (n=94), sampling adequacy for factor analysis assessed post hoc using the Kaiser-Meyer-Olkin (KMO) statistic to determine if criterion > 0.60 was met. Three factors explained a total of 84.9% of the variance: (a) Perceived Usefulness (b) Perceived Ease of Use and (c) Perceived Barriers to Use Behavioral Intention to Use was measured through a single item. | Both exploratory and confirmatory factor analysis yielded a one-factor solution. |

| | | | |
|---|---|---|---|
| Sample | Laypersons (health information consumers), and 20 experts (co-authors) from McGill University, and 3 organizational partners (Canadian Pharmacists Association , College of Family Physicians of Canada, Centre for Literacy Québec) | Case managers who provide care to persons living with HIV | Older adults aged 65-90 years |
| Limitations | Lay participants; convenience sample with adequate functional health literacy; needs further validation with a larger sample | Selection bias is possible since all participants in the study were Internet users and willing to complete and online survey, and so may be more likely to think favorably about technology. Respondents evaluated a mock-up of a CCR with context specific links, rather than fully functional systems, and did not use the system at the point of care delivery. | Authors state it was validated on a non-diverse sample that is relatively well-educated. |

| **Citation** | **Wang & Wang 2008** | **Wehmeyer 2008** | **Wolfradt & Doll, 2001** |
|---|---|---|---|
| Instrument name | Mobile Computing Self Efficacy | User-device Attachment | Internet Motivation Scale |
| Construct | Mobile computing self-efficacy (MCSE) defined as a summary judgment of one’s capability to engage in general and specific computing-related activities through a mobile computer | User-device attachment | Assesses three motives: information, interpersonal communication, and entertainment. |
| Theoretical foundation | Social cognitive theory, MCSE can be a significant antecedent of behavioral intention to use mobile computing. | Draws on scholarly work from marketing, sociology, information science, and human-computer interaction (HCI). | NR |

| | | | |
|---|---|---|---|
| Modification of another instrument | NA | No | NA |
| # items | 45 items (plus 6 global items) | 19 | 20 |
| Item types | Likert-type | Likert-type scale | 5-point Likert scale |
| Administration Time | NR | NR | NR |
| Item development | Developed based on the definition of MCSE and a review of the literature on computer self-efficacy, self-perceived computer competence, network competence, web-specific self-efficacy, and Internet self-efficacy. Reviewed with 2 information system professionals, two college teachers, and three mobile computer users. | Developed based on a review of the literature for each domain (symbolism, aesthetics, and perceived necessity) and subsequent group discussions with academic staff and IS and business students. Reviewed literature from marketing on material possession attachment, HCI research on the perception of aesthetics of physical and technical artifacts, and studies from sociology on cell phone usage. | NR |
| Scoring | Likert scale items are summed | NR | NR |
| Readability | NR | NR | NR |
| Sensitivity to change | NR | NR | NR |
| Reliability: test-retest | NR | NR | NR |
| Reliability: inter-rater | NR | NR | NR |
| Reliability: internal consistency | Cronbach's alpha for the MCSE =0.98 (then each factor: using basic mobile computer operations [.93], general use of the Internet [0.97], using e-mail [0.97], using specific mobile services [0.96], accessing/understanding mobile computer knowledge [0.94] | Cronbach's alpha =0.782 for symbolism, 0.860 for aesthetics, and 0.857 for necessity. | The internal consistencies (alpha) of the three motives were: 0.84 for information; 0.81 for interpersonal communication; and 0.76 for entertainment |

| | | | |
|---|---|---|---|
| Validity: content | The authors note that the rigorous procedures used to conceptualize the construct, and generate items representing the construct. | Preliminary items were reviewed with three expert judges (researchers in marketing and IS). | NR |
| Validity: criterion, convergent, concurrent, discriminant | Criterion-related validity assessed by the correlation between the total scores on the MCSE instrument and the 6 global measures of criterion (Cronbach's alpha=0.966); criterion-related validity=0.83 , p<0.001<br>Nomological validity evaluated by testing the hypothesis that there is a positive correlation between the MCSE score and the intention to use mobile computing (r=0.588, p<0.001) | Convergent validity ranged from 0.27 to 0.50 suggesting that the factors are not orthogonal.<br>Discriminant validity demonstrated by correlations not equal or close to 1.00 and low cross-loadings in the inter-subscale correlations. | Entertainment motive was positively associated with neuroticism and with all three personal factors (attitude, self-efficacy, innovativeness) but unrelated to the social factors<br>Interpersonal communication motive is positively related to neuroticism and extraversion, to self-efficacy, and to expectations of one's peer group to use the Internet. |
| Validity: construct | Factor analysis resulted in 5 factors, explained 77% of the variation (using basic mobile computer operations, general use of the Internet, using e-mail, using specific mobile services, accessing/understanding mobile computer knowledge) | Exploratory factor analysis using principal components analysis (n=130) followed by confirmatory analysis (n=232) resulting in three factors; explained 60% of variation.<br>Confirmatory factor analysis (n=323); | The three motives (information, communication, and entertainment) were associated with ways of reported internet use as expected (e.g., playing computer games is significantly associated with the entertainment motive, visiting chat rooms with the information (negative association) and communication (positive association) motives). |
| Sample | Employed adults in Taiwan; 60% male; age range 18 to 52 years | University employees and college students | German high school students ranging in age from fifteen to seventeen years who used the Internet on the average once a week |

| | | | |
|---|---|---|---|
| Limitations | Authors note that while it can be used to assess an individual's self-efficacy in mobile computing, a better way is to assess norms and then compare individual level with those norms | More work is needed to corroborate the initial conceptualization.<br>Testing performed in non-probabilistic samples may bias the results and may not be generalizable.<br>Snowball sampling used for confirmatory analysis may lead to respondent driven sampling.<br>Nomological validity as not been established yet.<br>The use of subjective scales and self-reports may inflate correlations due to common method bias. | Needs to be tested in other populations |

| **Citation** | **Xie, et al., 2013** | **Yip et al., 2003** |
|---|---|---|
| Instrument name | Health Information Wants Scale | No name |
| Construct | Patients' preferences for the (1) amount of information desired about different aspects of a health condition (Information Preference Scale), and (2) level of decision-making autonomy desired across those same aspects (Decision Making Preference Scale). | Satisfaction with telemedicine |
| Theoretical foundation | The Health Information Wants (HIW) framework | NR |
| Modification of another instrument | Yes | NR |
| # items | 21 for each scale | 14 |
| Item types | Likert-type (for the scales); multiple choice, open-ended | Likert-type |
| Administration Time | 15-45 minutes | NR |
| Item development | Previous literature and author's previous studies | Review of literature |
| Scoring | Calculate subscale scores and overall dimension scores as means across relevant items. | NR |
| Readability | NR | NR |
| Sensitivity to change | NR | NR |
| Reliability: test-retest | NR | NR |

| | | |
|---|---|---|
| Reliability: inter-rater | NR | NR |
| Reliability: internal consistency | Cronbach alpha coefficients ranged from .95-.71 for the younger age group, and .98-.78 for the older age group | Internal consistency 0.93 (did not specify Cronbach's alpha); item total correlation >0.3; intraclass correlation coefficient 0.43 |
| Validity: content | NR | Review by 14 physicians, nurses, and telemedicine experts |
| Validity: criterion, convergent, concurrent, discriminant | NR | Correlation between TSQ and self reported adherence significant ($r=0.45$, $p<0.05$) |
| Validity: construct | Confirmatory factor analysis supported construct validity | Factor analysis resulted in 4 factors; one eliminated; explained 68% of variation |
| Sample | College students; older adults recruited from senior centers | Chinese sample with diabetes |
| Limitations | Convenience sample | Validated in Chinese sample, needs further testing in other populations; needs convergent and divergent validity testing |
